# Evaluating short-term and long-term liver fibrosis improvement in hepatitis C patients after DAA treatment

**DOI:** 10.7555/JBR.37.20230284

**Published:** 2024-03-21

**Authors:** Yifan Wang, Xinyan Ma, Yanzheng Zou, Ming Yue, Meiling Zhang, Rongbin Yu, Hongbo Chen, Peng Huang

**Affiliations:** 1 Department of Infectious Disease, Jurong Hospital Affiliated to Jiangsu University, Jurong, Jiangsu 212400, China; 2 Department of Epidemiology, Center for Global Health, School of Public Health, Nanjing Medical University, Nanjing, Jiangsu 211166, China; 3 Department of Infectious Diseases, the First Affiliated Hospital of Nanjing Medical University, Nanjing, Jiangsu 210029, China

**Keywords:** chronic hepatitis C, sustained virologic response, direct-acting antivirals, liver fibrosis

## Abstract

Despite achieving a high cure rate with the direct-acting antivirals (DAAs) in hepatitis C treatment, further research is needed to identify additional benefits of the DAA therapy. The current study evaluated liver fibrosis improvement in 848 hepatitis C patients treated with DAAs, who also achieved sustained virologic response. By the fibrosis-4 (FIB-4) index, patients were categorized based on their baseline fibrosis levels, and the improvement in fibrosis was analyzed in both short-term (9–26 weeks) and long-term (≥ 36 weeks) follow-up. The results showed a significant decrease in the FIB-4 index, indicating an improvement in liver fibrosis, in 63.0% and 67.6% of the patients during the short-term and long-term follow-up, respectively. Short-term improvement was associated with factors including ribavirin usage, blood cholinesterase levels, alanine transaminase levels, albumin levels, and the baseline FIB-4 index, while long-term improvement was associated with factors such as aspartate transaminase levels, total protein level, and the baseline FIB-4 index. The current study emphasizes the importance of continuous assessment and post-treatment monitoring of liver fibrosis, which will provide crucial insights for enhancing patient care in hepatitis C management.

## Introduction

Hepatitis C virus (HCV) infection remains a significant global public health concern, affecting over 58 million individuals and contributing to 1.5 million new infections annually^[1]^. HCV infection progresses to a chronic state in approximately 55% to 85% of infected patients, potentially resulting in varying degrees of liver fibrosis^[2]^. Liver fibrosis significantly increases the risk of liver decompensation and hepatocellular carcinoma (HCC)^[3]^. Effective treatments are available for patients with hepatitis C. In May 2011, the first direct-acting antiviral (DAA) was approved by the U.S. Food and Drug Administration for the treatment of chronic hepatitis C^[4]^.

The efficacy of DAA therapy is influenced by the HCV genotype or subtypes, particularly because of the presence of resistance-associated substitutions at specific amino acid positions. Genotype identification through the NS5B sequence analysis may guide the tailoring of appropriate DAA regimens for different genotypes^[5]^. Generally, patients undergoing DAA treatment achieve high sustained virologic response (SVR) rates, often exceeding 95%^[6]^, including various patient subgroups, such as older individuals and those with cirrhosis, as the real-world data suggest that SVR rates are also very high in these individuals^[7]^.

Nevertheless, further research is warranted to explore additional benefits of DAA treatment for patients with hepatitis C. Recent studies investigated the changes in the degree of liver fibrosis in the patients after the DAA treatment^[8–9]^. However, these studies did not conduct separate analyses on patients with varying baseline liver fibrosis levels^[10]^. Moreover, the degree of liver fibrosis in the patients may change over time, and the extent of fibrosis changes at different time points after the treatment should be further investigated. The fibrosis-4 (FIB-4) index serves as a biomarker designed for evaluating liver fibrosis, and its cutoff values effectively classify the patients based on their degree of fibrosis^[11]^. Additionally, the FIB-4 index incorporates easily measurable parameters such as aspartate aminotransferase (AST), alanine aminotransferase (ALT), platelet count, and age^[12]^, making it easy to use for repeated measurements. Therefore, the FIB-4 index may be conveniently used to assess liver fibrosis in the patients at multiple time points. Furthermore, multiple factors are associated with the progression of liver fibrosis. A study conducted on HCV-infected individuals has demonstrated that hepatic steatosis and a history of diabetes mellitus contribute to the progression of liver fibrosis^[13]^. Factors such as sex^[14]^ and hypertension^[15]^ may also influence the progression of liver fibrosis.

To address these critical questions, we conducted a cohort study by stratifying the patients based on their initial FIB-4 index at the start of treatment. We aimed to study changes in liver fibrosis among patients with varying degrees of the condition. Subsequently, we assessed the FIB-4 index at different time points after the treatment to examine both short-term and long-term changes in liver fibrosis among the patients who achieved SVR with the DAA treatment. We also determined the factors associated with short-term and long-term improvements in liver fibrosis. The current study is expected to offer some novel insights into the post-DAA treatment monitoring of liver fibrosis.

## Subjects and methods

### Patients

A total of 1194 patients diagnosed with chronic HCV infection were enrolled in the Chronic Hepatitis C Research Program of Jiangsu (CHCRPJ) and received the DAA treatment at Jurong People's Hospital, China, between January 2012 and August 2022. The current study included patients aged 18 and older who received an all-oral DAA regimens and underwent HCV treatment following the local standard of care. Exclusion criteria included a history of liver transplantation, dialysis, or a pre-existing diagnosis of HCC before treatment initiation. Patients who did not achieve SVR after the DAA treatment were also excluded. The SVR was defined as a serum HCV RNA viral load below the lower limit of detection at least 12 weeks post treatment. A written informed consent was obtained from all participating patients, and the study protocol adhered to the ethical guidelines of the Declaration of Helsinki and was approved by the Institutional Review Board of Nanjing Medical University (Approval No. 528/2019).

### FIB-4 index

The baseline FIB-4 index was calculated using the laboratory test results obtained at the start of treatment, according to the following formula: (age × AST)/(platelet count × $ \sqrt{\mathrm{A}\mathrm{L}\mathrm{T}} $)^[16]^. AST was in U/L, platelet count was in 10^9^/L, and ALT was in U/L. Throughout the follow-up period, the FIB-4 index was recalculated based on laboratory test results collected during each patient's follow-up visit. Patients were categorized into three groups based on their FIB-4 index: < 1.45, 1.45–3.25, and > 3.25, which have been previously recognized as representing different degrees of liver fibrosis. An FIB-4 index below 1.45 was associated with a low likelihood of advanced fibrosis, while an FIB-4 index above 3.25 indicated a high probability of advanced fibrosis^[17]^. We considered a decrease of at least one point in the FIB-4 index as a clinically significant improvement in liver fibrosis, because with each unit increased in the FIB-4 index, the risk of developing severe liver disease also increased^[18]^.

### The short-term and long-term changes in the FIB-4 index of patients following treatment

For patients with a sufficient follow-up duration, the laboratory data required for the FIB-4 index were computed from baseline to both short-term and long-term follow-up time points. Short-term follow-up was defined as the last available value recorded between 9 and 26 weeks after the end of treatment, and long-term follow-up was defined as the last available value recorded at least 36 weeks after the end of treatment^[19]^. At the end of the follow-up period, a comparison of overall changes in the FIB-4 index with baseline was made. Patients were categorized into two groups based on their FIB-4 index: < 1.45 and ≥ 1.45. Subsequently, the incidence of HCC in each group was estimated by obtaining information about its diagnosis from hospital inpatient and outpatient records, following the guidelines outlined by the American Association for the Study of Liver Diseases (AASLD)^[20]^.

### Baseline variables

Baseline characteristics were collected at the initiation of treatment, including sex, age, cirrhosis, hypertension, diabetes, ribavirin (RBV) usage, creatinine, total bilirubin, direct bilirubin, ALT, AST, cholinesterase, alkaline phosphatase (ALP), gamma-glutamyl transferase (GGT), total protein, albumin, hemoglobin (HGB), platelet count, white blood cells (WBC), alpha-fetoprotein (AFP), blood urea, blood glucose (BG), total bile acid (TBA), triglycerides (TG), total cholesterol (T-Chol), high-density lipoprotein cholesterol (HDL-C), low-density lipoprotein cholesterol (LDL-C), apolipoprotein A1 (ApoA1), and apolipoprotein B (ApoB). Sex and age information was extracted from identity profiles, while cirrhosis, hypertension, diabetes, and RBV usage information was obtained from inpatient and outpatient diagnoses at the treatment initiation.

Cirrhosis was defined by biopsy (METAVIR stage 4 fibrosis) or FibroScan (liver stiffness of ≥ 12.5 kPa) and/or a combination of clinical, laboratory, histologic, and imaging criteria at the time of enrollment. To potentially enhance efficacy, clinicians may opt for a combination therapy with RBV depending on the patient's condition; hence, data on RBV usage were also collected. These variables were selected based on their availability in the current study and their associations with liver fibrosis, as described in literature^[21]^.

### Statistical analysis

Continuous variables were presented as median and interquartile range, and categorical variables were presented as counts and percentages. The Chi-square test or Fisher's exact test was performed to compare categorical variables when appropriate. Univariable and multivariable logistic regression models were performed to estimate associations of baseline variables with the short-term and long-term improvement in liver fibrosis. Variables that demonstrated statistical significance in univariable analysis (*P* < 0.05) were included in the multivariable analysis. Statistical significance was defined as *P* < 0.05. Data analysis was conducted using R software (version 4.1.2, R Foundation for Statistical Computing)^[22]^.

## Results

### Baseline characteristics of patients

Since January 2012, a total of 1194 patients with hepatitis C underwent treatment at Jurong People's Hospital. During the study period, some patients were excluded because of loss to the follow-up or withdrawal (*n* = 214), failure in the DAA treatment (*n* = 7), unavailable end-of-treatment records (*n* = 88), and unavailable baseline data (*n* = 37). The remaining 848 patients who achieved SVR constituted the study population. The median age of the patients was 58 (interquartile range [IQR], 52–63) years, with females accounting for 73% of the cohort. The median follow-up time was 155 weeks, and the longest follow-up period extended to 11.6 years, as detailed in ***Table 1***.

**Table 1:** Baseline characteristics of all patients and by FIB-4 index

Characteristics	Total (*n*=848)	FIB-4 (<1.45) (*n*=131)	FIB-4 (1.45–3.25) (*n*=372)	FIB-4 (>3.25) (*n*=345)
Age (years)	58 (52, 63)	51 (47, 56)	51 (47, 56)	61 (56, 66)
Female	623 (73%)	91 (69%)	268 (72%)	264 (77%)
Cirrhosis				
0	694 (82%)	125 (95%)	346 (93%)	223 (65%)
Compensated	101 (12%)	5 (4%)	19 (5%)	77 (22%)
Decompensated	53 (6%)	1 (1%)	7 (2%)	45 (13%)
Hypertension	154 (18%)	15 (11%)	73 (20%)	66 (19%)
Diabetes	85 (10%)	16 (12%)	34 (9%)	35 (10%)
RBV	337 (40%)	49 (37%)	170 (46%)	118 (34%)
FIB-4 index	2.79 (1.78, 4.54)	1.19 (1.04, 1.34)	2.16 (1.83, 2.73)	5.03 (3.98, 7.66)
Creatinine (μmol/L)	67 (57, 77)	66 (57, 78)	68 (58, 76)	66 (58, 77)
Total bilirubin (μmol/L)	15 (11, 20)	13 (10, 17)	15 (11, 19)	17 (13, 22)
Direct bilirubin (μmol/L)	4.15 (2.70, 6.10)	2.80 (2.00, 4.00)	3.80 (2.40, 5.30)	5.30 (3.50, 7.90)
ALT (U/L)	39 (23, 75)	30 (20, 49)	35 (21, 68)	51 (27, 97)
AST (U/L)	36 (25, 60)	24 (20, 34)	31 (23, 50)	51 (33, 83)
Cholinesterase (U/L)	6835 (5634, 7955)	7990 (6830, 9407)	7216 (6202, 8191)	5895 (4737, 7033)
ALP (U/L)	75 (61, 94)	67 (56, 82)	69 (57, 86)	86 (70, 108)
GGT (U/L)	35 (22, 64)	26 (18, 43)	29 (19, 51)	47 (28, 82)
Total protein (g/L)	79 (75, 83)	79 (75, 82)	79 (75, 82)	80 (75, 84)
Albumin (g/L)	45.3 (42.3, 47.9)	46.5 (43.6, 49.0)	45.6 (42.9, 48.2)	44.2 (41.3, 46.9)
HGB (g/L)	132 (120, 142)	135 (122, 147)	133 (122, 142)	129 (116, 140)
Platelet count (10^9^/L)	125 (87, 165)	199 (175, 221)	140 (120, 165)	81 (62, 105)
WBC (10^9^/L)	5 (4, 6)	6 (5, 8)	5 (4, 7)	4 (3, 5)
AFP (ng/mL)	4 (3, 6)	4 (2, 4)	4 (3, 5)	5 (4, 10)
Urea (mmol/L)	4.50 (3.59, 5.35)	4.39 (3.58, 5.27)	4.55 (3.59, 5.32)	4.45 (3.54, 5.47)
BG (mmol/L)	5.73 (5.19, 5.97)	5.69 (5.14, 5.84)	5.70 (5.21, 5.86)	5.78 (5.18, 6.18)
TBA (μmol/L)	7 (4, 10)	5 (2, 7)	6 (3, 8)	7 (5, 14)
TG (mmol/L)	1.14 (0.96, 1.38)	1.15 (1.04, 1.58)	1.14 (0.99, 1.36)	1.14 (0.93, 1.31)
T-Chol (mmol/L)	4.32 (3.88, 4.72)	4.38 (4.19, 5.15)	4.32 (4.02, 4.77)	4.32 (3.75, 4.38)
HDL-C (mmol/L)	1.62 (1.38, 1.78)	1.62 (1.38, 1.83)	1.62 (1.38, 1.80)	1.62 (1.41, 1.73)
LDL-C (mmol/L)	2.51 (2.19, 2.72)	2.59 (2.42, 3.13)	2.51 (2.30, 2.79)	2.49 (2.03, 2.51)
ApoA1 (g/L)	1.65 (1.51, 1.79)	1.65 (1.51, 1.84)	1.65 (1.53, 1.79)	1.65 (1.48, 1.76)
ApoB (g/L)	0.76 (0.65, 0.86)	0.76 (0.67, 0.97)	0.76 (0.67, 0.89)	0.76 (0.60, 0.79)
Continuous variables were presented as median (interquartile range), and categorical variables were presented as count (percentage). Abbreviations: FIB-4, fibrosis-4; RBV, ribavirin; HCC, hepatocellular carcinoma; ALT, alanine aminotransferase; AST, aspartate aminotransferase; ALP, alkaline phosphatase; GGT, gramma-glutamyl transferase; HGB, hemoglobin; WBC, white blood cell; AFP, alpha-fetoprotein; BG, blood glucose; TBA, total bile acid; TG, triglycerides; T-Chol, total cholesterol; HDL-C, high-density lipoprotein cholesterol; LDL-C, low density lipoprotein cholesterol; ApoA1, apolipoprotein A1; ApoB, apolipoprotein B.				

Before the start of treatment, the median FIB-4 index of the patients was 2.79 (IQR, 1.78–4.54). Among the 848 patients, 131 patients (15.45%) had an FIB-4 index < 1.45, 372 patients (43.87%) had an FIB-4 index between 1.45 and 3.25, and 345 patients (40.68%) had an FIB-4 index > 3.25.

### DAA regimen distribution and RBV usage

Among the 848 patients, the most frequently prescribed DAA regimen was sofosbuvir/velpatasvir (34.91%), followed by sofosbuvir/daclatasvir (16.27%) and sofosbuvir/ledipasvir (6.37%). In patients with an FIB-4 index > 3.25, the predominant DAA regimen was sofosbuvir/velpatasvir (36.52%). Additionally, RBV was administered to 40% of the patients, with no significant differences observed in its usage among the FIB-4 groups.

### The short-term changes in liver fibrosis

A total of 572 patients had available FIB-4 index data between 9 and 36 weeks after the DAA treatment, of whom 200 patients (34.97%) exhibited an increase in their FIB-4 index, compared with the baseline, while 11 patients (1.92%) showed no change. In contrast, a decrease in the FIB-4 index was observed in 361 patients (63.11%), with an average decrease of 2.01. Among the patients who experienced a decrease, 37 patients had a baseline FIB-4 index < 1.45, 146 patients had a baseline FIB-4 index between 1.45 and 3.25, and 178 patients had a baseline FIB-4 index > 3.25.

In the univariable analysis, several factors were found to be associated with a short-term decrease in the FIB-4 index greater than one, including RBV usage, cirrhosis, baseline FIB-4 stage, total bilirubin levels, direct bilirubin levels, cholinesterase levels, ALP levels, GGT levels, albumin levels, HGB levels, AFP levels, TBA levels, T-Chol levels, LDL-C levels, and ApoB levels (***Supplementary Table 1***, available online). Conversely, sex, hypertension, and diabetes did not exhibit a significant association.

When the factors significant in the univariable analysis were included in the multivariable analysis, RBV usage, cholinesterase levels, GGT levels, albumin levels, and baseline FIB-4 stage remained significantly associated with the FIB-4 index improvement (***Table 2***).

**Table 2:** Multivariable analysis to predict improvement in FIB-4 index (decrease by over one point) from baseline to 9–26 weeks after DAA treatment

Characteristics	Univariable			Multivariable	
	OR (95% CI)	*P*-value		OR (95% CI)	*P*-value
RBV	0.49 (0.33, 0.73)	< 0.001		0.58 (0.34, 0.97)	0.039
Cirrhosis	8.61 (5.40, 14.21)	< 0.001		0.77 (0.35, 1.61)	
Total bilirubin (μmol/L)	1.03 (1.01, 1.05)	< 0.001		1.03 (0.98, 1.08)	
Direct bilirubin (μmol/L)	1.08 (1.03 , 1.13)	0.001		0.96 (0.89, 1.03)	
Cholinesterase (U/L)	0.99 (0.99, 0.99)	< 0.001		0.99 (0.99, 0.99)	0.024
ALP (U/L)	1.01 (1.01, 1.02)	< 0.001		1.00 (0.99, 1.01)	
GGT (U/L)	1.01 (1.00, 1.01)	< 0.001		1.01 (1.00, 1.01)	0.033
Albumin (g/L)	0.95 (0.91, 0.99)	0.023		1.08 (1.01, 1.15)	0.033
HGB (g/L)	0.99 (0.98, 1.00)	0.005		0.99 (0.98, 1.01)	
AFP (ng/mL)	1.01 (1.00, 1.03)	0.038		1.00 (0.98, 1.01)	
TBA (μmol/L)	1.02 (1.01, 1.04)	< 0.001		1.00 (0.98, 1.02)	
T-Chol (mmol/L)	0.62 (0.47, 0.80)	< 0.001		1.41 (0.77, 2.58)	
LDL-C (mmol/L)	0.35 (0.23, 0.52)	< 0.001		0.46 (0.18, 1.16)	
ApoB (g/L)	0.36 (0.14, 0.91)	0.034		0.96 (0.20, 4.53)	
Baseline FIB-4 stage	21.67 (12.33, 41.41)	< 0.001		16.93 (7.99, 39.47)	< 0.001
Abbreviations: DAA, direct-acting antiviral; OR, odds ratio; RBV, ribavirin; ALP, alkaline phosphatase; GGT, gramma-glutamyl transferase; HGB, hemoglobin; AFP, alpha-fetoprotein; TBA, total bile acid; T-Chol, total cholesterol; LDL-C, low density lipoprotein cholesterol; ApoB, apolipoprotein B.					

### The long-term changes in liver fibrosis

In the population of the current study, 562 patients who achieved SVR at baseline were included for the evaluation of long-term liver fibrosis. Among these 562 patients, 178 patients (31.67%) demonstrated an increase in the FIB-4 index, while only five patients (0.89%) exhibited no change. Conversely, 379 patients (67.44%) showed a decrease in the FIB-4 index, with an average reduction of 1.8. Among these 379 patients, 31 had a baseline FIB-4 index < 1.45, 175 had a baseline FIB-4 index between 1.45 and 3.25, and 173 had a baseline FIB-4 index > 3.25.

In the univariable analysis, the factors associated with a long-term decrease in the FIB-4 index greater than one included RBV usage, cirrhosis, baseline FIB-4 stage, total bilirubin levels, direct bilirubin levels, cholinesterase levels, ALP levels, GGT levels, total protein levels, albumin levels, TBA levels, T-Chol levels, HDL-C levels, LDL-C levels, and ApoB levels (***Supplementary Table 2***, available online).

In the multivariable analysis, GGT levels, total protein levels, and baseline FIB-4 stage remained significantly associated with a long-term decline by greater than one in the FIB-4 index (***Table 3***).

**Table 3:** Multivariable analysis to predict improvement in FIB-4 index (decrease by over one point) from baseline to ≥ 36 weeks after DAA treatment

Characteristics	Univariable			Multivariable	
	OR (95% CI)	*P*-value		OR (95% CI)	*P*-value
RBV	0.68 (0.48, 0.97)	0.035		0.94 (0.58, 1.52)	
Cirrhosis	7.38 (4.96, 11.16)	< 0.001		0.85 (0.42, 1.65)	
Direct bilirubin (μmol/L)	1.18 (1.12, 1.25)	< 0.001		0.95 (0.87, 1.07)	
Total bilirubin (μmol/L)	1.06 (1.04, 1.18)	< 0.001		1.05 (1.00, 1.11)	
Cholinesterase (U/L)	0.99 (0.99, 0.99)	< 0.001		1.00 (0.99, 1.00)	
ALP (U/L)	1.02 (1.00, 1.02)	< 0.001		1.00 (0.99, 1.01)	
GGT (U/L)	1.01 (1.00, 1.01)	< 0.001		1.00 (1.00, 1.01)	0.045
Total protein (g/L)	1.03 (1.01, 1.06)	0.017		1.06 (1.02, 1.10)	0.002
Albumin (g/L)	0.92 (0.89, 0.96)	< 0.001		0.95 (0.89, 1.02)	
TBA (μmol/L)	1.02 (1.01, 1.04)	< 0.001		0.99 (0.97, 1.00)	
T-Chol (mmol/L)	0.61 (0.47, 0.77)	< 0.001		0.73 (0.37, 1.43)	
HDL-C (mmol/L)	0.50 (0.29, 0.82)	0.009		0.86 (0.37, 1.90)	
LDL-C (mmol/L)	0.41 (0.28, 0.58)	<0.001		0.70 (0.27, 1.73)	
ApoB (g/L)	0.39 (0.17, 0.91)	0.032		3.92 (0.79, 19.17)	
Baseline FIB-4 stage	12.12 (8.09, 16.69)	<0.001		10.76 (5.75, 20.12)	<0.001
Abbreviations: DAA, direct-acting antiviral; OR, odds ratio; RBV, ribavirin; ALP, alkaline phosphatase; GGT, gramma-glutamyl transferase; HGB, hemoglobin; TBA, total bile acid; T-Chol, total cholesterol; HDL-C, high-density lipoprotein cholesterol; LDL-C, low-density lipoprotein cholesterol; ApoB, apolipoprotein B.					

### Liver fibrosis status at the end of follow-up

At the end of follow-up, the last admitted patients had also surpassed 36 weeks from the start of treatment. Compared with the baseline, there was an average decrease of 0.88 in the FIB-4 index. During the follow-up period, 40 patients developed HCC. We divided the patients at the end of follow-up into two groups based on their FIB-4 index: the FIB-4 index < 1.45 group consisting of 135 patients, and the FIB-4 index ≥ 1.45 group consisting of 427 patients. Out of the 40 HCC cases, three cases occurred in the FIB-4 index < 1.45 group, while the other 37 cases occurred in the FIB-4 index ≥ 1.45 group.

## Discussion

Because of the high SVR rates achieved with the DAA therapy, the DAA treatment is recommended for nearly all stages of HCV infection^[8]^. Although some studies have investigated changes in the degree of liver fibrosis following the DAA treatment^[8–9]^, there is limited research categorizing patients based on different levels of liver fibrosis and subsequently describing fibrosis changes within each subgroup. Moreover, the status of liver fibrosis in the patients may evolve, and there is also limited research on the changes in liver fibrosis status at different time points after the DAA treatment. Therefore, a study focusing on patients with different degrees of liver fibrosis and their fibrosis changes at various time points after the DAA treatment is needed to address these gaps.

In the current study, we conducted a real-world, prospective cohort study analysis that focused on the DAA treatment. The cohort included 848 patients who received the DAA treatment and achieved SVR, with some patients being followed up for over 10 years. Patients were divided into three subgroups based on the FIB-4 index and both long-term and short-term changes in liver fibrosis were observed. Additionally, univariable and multivariable logistic regression analyses were performed to investigate the factors influencing the short-term and long-term improvement in liver fibrosis after the DAA treatment.

Firstly, our study observed that liver fibrosis improved significantly in the short term in most patients after the DAA treatment. Approximately 63.1% of the patients showed a decrease in their FIB-4 index in the short term, with an overall average decrease of 0.59. In 24.6% of the patients, the FIB-4 index decreased by more than one. Furthermore, the results from the long-term analysis revealed that 67.4% of the patients showed a decrease in their FIB-4 index, with an overall average decrease of 0.88. Additionally, 33.7% of the patients showed a decrease of more than one in their FIB-4 index. A previous multicenter observational study conducted on patients with hepatitis C across four U.S. health systems also documented a substantial decrease in the FIB-4 index among patients following the DAA therapy^[23]^. This decrease remained significant among those who achieved SVR over 10 years, compared with the untreated patients or those with treatment failure^[24]^.

Secondly, our univariable analysis revealed significant associations between certain baseline variables and changes in liver fibrosis. Specifically, we observed that age and AFP levels were associated with short-term improvement in liver fibrosis. Feng *et al*^[25]^ developed a non-invasive index for predicting significant fibrosis, which included AFP with a good predictive performance. Both age and elevated AFP are associated with some biological processes, such as inflammation, injury, and regeneration, which in turn influence the course of fibrosis. The current study also demonstrated that T-Chol, LDL-C, and ApoB levels were associated with both short-term and long-term improvements in liver fibrosis. Previous research has suggested that HCV infection may have early negative effects on lipid metabolism; however, the HCV eradication obtained by DAA may eliminate these negative effects^[26]^. Additionally, HCV competes with the liver's LDL-C receptor, possibly leading to hypobetalipoproteinemia^[27]^, which may result in steatosis in patients with chronic hepatitis C. The interaction between HCV infection and lipid metabolism may partially explain our findings.

Finally, we performed a multivariable analysis based on the results of the univariable analysis. As expected, we found that both short-term and long-term improvements in liver fibrosis after the treatment were significantly associated with the baseline liver fibrosis stage. One study reported that patients with advanced liver fibrosis might experience a relatively slower recovery and that some individuals might still exhibit elevated levels of liver fibrosis even after successful eradication of HCV following the initiation of DAA treatment^[11]^. Our results also demonstrated that RBV usage was significantly associated with short-term liver fibrosis improvement. RBV has some known side effects, including anemia and other blood-related issues^[28–29]^, which may affect short-term liver fibrosis changes. In addition, we also observed that levels of albumin, GGT, and cholinesterase were associated with short-term liver fibrosis improvement. The finding that baseline albumin levels were associated with short-term improvement in liver fibrosis is similar to that of a previous report^[30]^, in which the authors observed an association between albumin levels and improvement in liver function among patients with cirrhosis. Moreover, another study utilizing ultrastructural observation to assess liver function suggested that elevated levels of cholinesterase and GGT might indicate severe liver fibrosis^[31]^. Notably, we also found an association between GGT levels and the long-term improvement in liver fibrosis. As a multifunctional protein, GGT has not only been associated with overall mortality in patients receiving the DAA-induced SVR^[32]^, but also been identified as an independent predictor of adverse clinical outcomes in individuals with advanced liver disease because of HCV^[33]^. These imply that patients with high GGT levels may have an unfavorable prognosis, potentially influencing the extent of improvement in liver fibrosis.

At the end of the follow-up, 24.02% of the patients had an FIB-4 index < 1.45. Among these patients, only three developed HCC, accounting for just 7.5% of all the HCC cases. The remaining 37 new cases were in the group with an FIB-4 index ≥ 1.45. Similar results were reported in previous studies. For example, one study investigated the long-term risk of HCC in patients who achieved SVR after treatment and found that the patients with a high FIB-4 index had a significantly higher incidence of HCC^[34]^. These findings suggest that clinicians should focus on patients with a high degree of post-treatment liver fibrosis levels, as they are at an increased risk of developing HCC.

The current study also observed an increased risk of HCC in patients with an FIB-4 index over 3.25 after the DAA treatment, consistent with previous reports of HCC recurrence post-DAA therapy^[35]^. This underscores the need for regular surveillance and screening, particularly in the patients with a high FIB-4 index. The AASLD recommends biannual HCC screening for all adults with cirrhosis^[36]^. The European Association for the Study of the Liver further extends this recommendation to include patients with advanced liver fibrosis (F3)^[37]^. The surveillance protocol primarily includes the use of ultrasound, optionally combined with AFP testing^[38]^. Early detection of HCC is particularly important, as it facilitates timely interventions that are crucial in improving treatment efficacy and enhancing overall patient survival outcomes.

In addition to monitoring for HCC, post-DAA surveillance should also include assessments for other comorbidities. One study reported a significant increase in serum neutrophil gelatinase-associated lipocalin, indicative of tubular damage, during the DAA treatment, suggesting an impact on renal function^[39]^. Similarly, another study found an unexpected worsening of left ventricular function, as measured by global longitudinal strain, after HCV treatment with DAAs^[40]^. Therefore, it is essential to encompass both cardiac and renal assessments in the long-term follow-up of HCV patients post-treatment for a more comprehensive analysis, particularly in those with a higher FIB-4 index.

The current study has several strengths. Firstly, all patients included in the study received the DAA treatment and achieved SVR. Secondly, the follow-up period spans over a decade in this cohort, providing a relatively long-term perspective. Previous studies examining the predictive value of the FIB-4 index were either conducted in patients receiving interferon treatment^[41]^ or lacked a sufficiently long follow-up period in the DAA-treated patients^[42]^. Finally, the current study classified the patients based on the FIB-4 index to focus on the changes in the degree of liver fibrosis in different subgroups and their changes at different time points after the treatment.

However, the current study also has several limitations. Firstly, as time progressed, some patients did not have regular follow-up examinations after achieving SVR, resulting in missing data. Secondly, the current study is a single-center study, and the generalizability of our results to a larger HCV population may be limited. Thirdly, the study cohort consists entirely of an Asian population and is predominantly female. Therefore, it is important to conduct validation studies in other cohorts to support these findings. Finally, it is acknowledged that the current study lacks data on alcohol consumption and body mass index—factors known to be associated with liver fibrosis. Future investigations may consider incorporating these variables for a more comprehensive analysis of liver fibrosis and its changes after the DAA treatment.

In addition to the FIB-4 index, we acknowledge the potential of other noninvasive methods, such as liver elastography, for a more comprehensive assessment of liver fibrosis. Yaraş *et al*^[43]^ used ultrasonography-based elastography to monitor liver stiffness in patients with hepatitis C undergoing the DAA treatment, and they observed a significant reduction in liver stiffness post-treatment, also consistent with the reduction in the FIB-4 index noted in their study. Unfortunately, we were unable to include liver stiffness data because of the unavailability of necessary equipment. Future studies should consider incorporating liver stiffness data measured by ultrasonography-based elastography, along with a longer period of follow-up, potentially yielding even more robust and insightful results.

In summary, the current study had several key findings. Firstly, we observed significant improvements in liver fibrosis using the FIB-4 index among most patients following SVR in both short-term and long-term follow-up. Secondly, baseline factors, such as RBV usage, cholinesterase levels, GGT levels, albumin levels, total protein levels, and baseline FIB-4 stage, were found to be associated with fibrosis improvement. Notably, regardless of the short-term or long-term time frame, different liver fibrosis stage groupings significantly influenced the improvement of patients' liver fibrosis. Finally, we revealed a significantly increased risk of HCC among the patients with a higher FIB-4 index. Consequently, we demonstrated the importance of surveillance in patients who showed higher levels of fibrosis after the DAA treatment, providing a valuable guidance for future research and clinical practice.

## SUPPLEMENTARY DATA

Supplementary data to this article can be found online.
