# Supplementary material for: Evaluating short-term and long-term liver fibrosis improvement in hepatitis C patients after DAA treatment
Source: J Biomed Res. 2024 Mar 21;38(5):464–72. doi: 10.7555/JBR.37.20230284 (PMC11461531; doi:10.7555/JBR.37.20230284)
Supplement: Supplementary file 1 — Supplementary data to this article can be found online. [file jbr-38-5-464-S1.pdf]

Available online at www.jbr-pub.org.cn

Open Access at PubMed Central

Journal of Biomedical Research, 2024 38(5): 1–2

Supplementary Data

JBR

Evaluating short-term and long-term liver fibrosis improvement in hepatitis C patients after DAA treatment

Yifan Wang[1,△], Xinyan Ma[2,△], Yanzheng Zou[2,△], Ming Yue[3], Meiling Zhang[1], Rongbin Yu[2], Hongbo Chen[1,✉], Peng Huang[2,✉]

[1]*Department of Infectious Disease, Jurong Hospital Affiliated to Jiangsu University, Jurong, Jiangsu 212400, China;*
[2]*Department of Epidemiology, Center for Global Health, School of Public Health, Nanjing Medical University, Nanjing, Jiangsu 211166, China;*
[3]*Department of Infectious Diseases, the First Affiliated Hospital of Nanjing Medical University, Nanjing, Jiangsu 210029, China.*

Supplementary Table 1 **Univariable analysis to predict improvement in FIB-4 (decrease by over one point) from baseline to 9–26 weeks after DAA treatment**

Characteristics	OR (95% CI)	*P*-value
Age (years)	1.09 (0.99, 1.19)	0.285
Sex	1.01 (0.79, 1.23)	0.960
Cirrhosis	8.61 (5.40, 14.21)	<0.001
Hypertension	0.81 (0.55, 1.07)	0.070
Diabetes	0.71 (0.36, 1.06)	0.336
RBV	0.49 (0.33, 0.73)	<0.001
Creatinine (mmol/L)	0.99 (0.99, 1.00)	0.817
Total bilirubin (μmol/L)	1.03 (1.01, 1.05)	<0.001
Direct bilirubin (μmol/L)	1.08 (1.03 , 1.13)	0.001
ALT (U/L)	1.01 (0.99, 1.01)	0.114
AST (U/L)	1.03 (0.99, 1.04)	0.867
Cholinesterase (U/L)	0.99 (0.99, 0.99)	<0.001
ALP (U/L)	1.01 (1.01, 1.02)	<0.001
GGT (U/L)	1.01 (1.00, 1.01)	<0.001
Total protein (g/L)	1.01 (0.99, 1.03)	0.417
Albumin (g/L)	0.95 (0.91, 0.99)	0.023
HGB (g/L)	0.99 (0.98, 1.00)	0.005
Platelet count (10^9/L)	0.97 (0.94, 1.00)	0.061
WBC (10^9/L)	0.99 (0.98, 1.01)	0.346
AFP (ng/ml)	1.01 (1.00, 1.03)	0.038
Urea (mmol/L)	1.05 (0.98, 1.11)	0.479
BG (mmol/L)	0.94 (0.88, 1.00)	0.327
TBA (μmol/L)	1.02 (1.01, 1.04)	<0.001

△These authors contributed equally to this work.

✉Corresponding authors: Hongbo Chen, Department of Infectious Disease, Jurong Hospital Affiliated to Jiangsu University, Jurong, Jiangsu 212400, China. E-mail: chb2180@126.com; Peng Huang, Department of Epidemiology, Center for Global Health, School of Public Health, Nanjing Medical University, Nanjing, Jiangsu 211166, China. E-mail: huangpeng@njmu.edu.cn.

Received: 23 November 2023; Revised: 20 January 2024; Accepted: 23 January 2024; Published online: 21 March 2024

CLC number: R512.63, Document code: A

The authors reported no conflict of interests.

This is an open access article under the Creative Commons Attribution (CC BY 4.0) license, which permits others to distribute, remix, adapt and build upon this work, for commercial use, provided the original work is properly cited.

© 2024 by Journal of Biomedical Research.

https://doi.org/10.7555/JBR.37.20230284

Supplementary Table 1 **Univariable analysis to predict improvement in FIB-4 (decrease by over one point) from baseline to 9–26 weeks after DAA treatment** (Continued)

Characteristics	OR (95% CI)	*P*-value
TG (mmol/L)	0.70 (0.39, 1.00)	0.096
T-Chol (mmol/L)	0.62 (0.47, 0.80)	<0.001
HDL-C (mmol/L)	0.81 (0.62, 1.00)	0.073
LDL-C (mmol/L)	0.35 (0.23, 0.52)	<0.001
ApoA1 (g/L)	0.74 (0.40, 1.08)	0.369
ApoB (g/L)	0.36 (0.14, 0.91)	0.034
Baseline FIB-4 stage	21.67 (12.33, 41.41)	<0.001

Abbreviations: DAA, direct-acting antiviral; OR, odds ratio; RBV, ribavirin; ALT, alanine aminotransferase; AST, aspartate aminotransferase; ALP, alkaline phosphatase; GGT, gamma-glutamyl transferase; HGB, hemoglobin; WBC, white blood cell; AFP, alpha-fetoprotein; BG, blood glucose; TBA, total bile acid; TG, triglycerides; T-Chol, total cholesterol; HDL-C, high-density lipoprotein cholesterol; LDL-C, low density lipoprotein cholesterol; ApoA1, apolipoprotein A1; ApoB, apolipoprotein B.

Supplementary Table 2 **Univariable analysis to predict improvement in FIB-4 (decrease by over one point) from baseline to ⩾ 36 weeks after DAA treatment**

Characteristics	OR (95% CI)	*P*-value
Age (years)	1.07 (0.97, 1.17)	0.343
Sex	1.09 (0.88, 1.30)	0.691
Hypertension	1.14 (0.92, 1.36)	0.539
Diabetes	1.28 (0.99, 1.57)	0.369
RBV	0.68 (0.48, 0.97)	0.035
Cirrhosis	7.38 (4.96, 11.16)	<0.001
Creatinine (mmol/L)	1.00 (0.99, 1.00)	0.921
Direct bilirubin (μmol/L)	1.18 (1.12, 1.25)	<0.001
Total bilirubin (μmol/L)	1.06 (1.04, 1.18)	<0.001
ALT (U/L)	1.01 (1.00, 1.01)	0.231
AST (U/L)	1.02 (0.99, 1.05)	0.283
Cholinesterase (U/L)	0.99 (0.99, 0.99)	<0.001
ALP (U/L)	1.02 (1.00, 1.02)	<0.001
GGT (U/L)	1.01 (1.00, 1.01)	<0.001
Total protein (g/L)	1.03 (1.01, 1.06)	0.017
Albumin (g/L)	0.92 (0.89, 0.96)	<0.001
HGB (g/L)	0.98 (0.94, 1.02)	0.685
Platelet count (10^9/L)	0.97 (0.95, 1.00)	0.054
WBC (10^9/L)	0.99 (0.99, 1.00)	0.477
AFP (ng/ml)	1.01 (1.00, 1.01)	0.145
Urea (mmol/L)	0.92 (0.83, 1.01)	0.153
BG (mmol/L)	1.04 (1.00, 1.07)	0.153
TBA (μmol/L)	1.02 (1.01, 1.04)	<0.001
TG (mmol/L)	1.08 (1.00, 1.16)	0.326
T-Chol (mmol/L)	0.61 (0.47, 0.77)	<0.001
HDL-C (mmol/L)	0.50 (0.29, 0.82)	0.009
LDL-C (mmol/L)	0.41 (0.28, 0.58)	<0.001
ApoA1 (g/L)	0.74 (0.41, 1.07)	0.067
ApoB (g/L)	0.39 (0.17, 0.91)	0.032
Baseline FIB-4 stage	12.12 (8.09, 16.69)	<0.001

Abbreviations: DAA, direct-acting antiviral; OR, odds ratio; RBV, ribavirin; ALT, alanine aminotransferase; AST, aspartate aminotransferase; ALP, alkaline phosphatase; GGT, gamma-glutamyl transferase; HGB, hemoglobin; WBC, white blood cell; AFP, alpha-fetoprotein; BG, blood glucose; TBA, total bile acid; TG, triglycerides; T-Chol, total cholesterol; HDL-C, high-density lipoprotein cholesterol; LDL-C, low density lipoprotein cholesterol; ApoA1, apolipoprotein A1; ApoB, apolipoprotein B.
